# Human-taught sensory-control synergy for universal robotic grasping

**DOI:** 10.1093/nsr/nwaf583

**Published:** 2025-12-22

**Authors:** Caise Wei, Zijian Liao, Yichen Qin, Qian Mao, Shiqiang Liu, Rong Zhu

**Affiliations:** State Key Laboratory of Precision Measurement Technology and Instrument, Department of Precision Instrument, Tsinghua university, Beijing 100084, China; State Key Laboratory of Precision Measurement Technology and Instrument, Department of Precision Instrument, Tsinghua university, Beijing 100084, China; State Key Laboratory of Precision Measurement Technology and Instrument, Department of Precision Instrument, Tsinghua university, Beijing 100084, China; State Key Laboratory of Precision Measurement Technology and Instrument, Department of Precision Instrument, Tsinghua university, Beijing 100084, China; State Key Laboratory of Precision Measurement Technology and Instrument, Department of Precision Instrument, Tsinghua university, Beijing 100084, China; State Key Laboratory of Precision Measurement Technology and Instrument, Department of Precision Instrument, Tsinghua university, Beijing 100084, China

**Keywords:** universal robotic grasping, sensory-control synergy, multimodal tactile sensor, human skill transfer

## Abstract

Universal grasping is essential but challenging in robotic manipulations, particularly for humanoid robots with multifingered hands. To learn skills of dexterous manipulations from humans, we propose a sensory-control synergy (SCS) approach mimicking the human grasping experience. We develop a tactile glove worn on a human hand to capture multimodal tactile data (contact, slip and pressure) during human grasping demonstrations. Emulating human neurocognition, the multimodal tactile data are encoded into semantically explicit grasping states by neural-network computing. Drawing on human motor control strategies, an experience-based fuzzy controller is built to swiftly convert semantic grasping states into grasping actions. Benefiting from the semantization of grasping states, the SCS model is highly logicalized and generalizable, can be data-efficiently built by non-experts and readily transferred to robots for accomplishing universal robotic manipulation. The robot with SCS achieves an average success rate of 95.2% in grasping diverse objects of daily life, including slippery, fragile, soft and heavy objects. In dynamic disturbance and complex tasks, the robot autonomously manipulates using its adaptive SCS, demonstrating human-like universal grasping capabilities.

## Teaser

Human-like sensory-control synergy (SCS) approach enables robots to data-efficiently learn skills of dexterous manipulations from humans, achieving adaptive and universal grasping diverse objects of daily life.

## INTRODUCTION

Grasping capability, as a foundational skill of robots interacting with physical environments, determines the intelligence level of robots in open-world scenarios [1–3]. Early robotic grasping primarily relied on pre-programmed target information or constant force control strategies to accomplish simple tasks through mechanically repetitive fixed actions. Such methods lack adaptability to unstructured environments, making it difficult to generalize to complex scenarios with diverse objects. As service and industrial robots penetrate increasingly complex environments (e.g. home assistance, flexible manufacturing), endowing robots (especially humanoid robots with multifingered dexterous hands) with universal grasping capabilities has emerged as a core challenge in embodied intelligence and robotic manipulation [4].

For robot perception, early studies typically adopted cameras to capture object pose and grasp-point data [5–8]. For instance, publicly available datasets such as the Cornell Grasp Dataset and the Cross-Background Robot Grasp Detection Dataset support training of 2D/3D grasp detection models through RGB-D images and annotated grasp rectangles [9,10]. Nevertheless, visual perception fails to capture the detailed interactions in grasping, leading to unintended physical outcomes, such as the object sliding off and crushing. The tactile senses can provide a crucial complement to address the issues [11,12]. The tactile sensors capture the interaction data, including contact forces, surface textures and slip events in real time, enabling robots to dynamically adjust grasping forces, which potentially adapts to diverse objects with different attributes, reduces the risk of damage or dropping, and increases the grasping success rates [13–15]. Consequently, visual–tactile fusion has become the dominant paradigm for enhancing grasping success rates [16–18]. Vision provides target localization and environment awareness, while tactile sensing delivers delicate interaction responses and fine-grained compliance control. Their synergistic integration significantly improves task accomplishment rates in cluttered and dynamic scenarios [19].

Despite efficient utilization of visual data due to abundant internet resources (e.g. ImageNet, COCO) and mature pre-trained models (e.g. CLIP), the construction of tactile datasets remains a formidable challenge. Current approaches for acquiring tactile datasets primarily fall into three categories: simulation-based data generation [20,21]; robot teleoperation collection [22]; and the emerging paradigm of wearable devices [23–25]. Simulation-based data generation methods synthesize large-scale grasping data through the finite element method (FEM) or physics engines (e.g. NVIDIA Omniverse). However, they exhibit significant deviations in modeling physical properties of non-rigid objects (e.g. silicone toys, food items), leading to drastic performance degradation in simulation-to-reality (Sim2Real) transfer [26,27]. The robot teleoperation collection approach records real interaction data by remotely controlling robotic arms [28]. Although this method potentially collects high-fidelity data, it relies on specialized hardware and suffers from low collection efficiency, making it difficult to cover diverse objects and complex scenarios. Recently, learning manipulation skills from human demonstration has been considered an effective way to expand robotic capabilities. Tactile gloves or hand exoskeleton systems are developed to construct datasets. These systems record human hand motion trajectories and tactile signals during manipulation [29–31]. However, due to fundamental differences in the structure and function of ontology between humans and robots, directly replicating human behaviors is often infeasible, presenting significant challenges for skill transfer.

In recent years, multimodal grasping models adopting end-to-end deep learning architectures have been proposed, which directly map raw sensory inputs to final control parameters such as grasping poses or force commands [32,33]. This paradigm simplifies the data processing pipelines, but the model’s generalization heavily relies on massive annotated datasets. Moreover, these models become prone to overfitting with limited samples and struggle to reveal the causal relationships between tactile signals and control strategies, resulting in fragile strategies and difficult debugging processes. Consequently, researchers are gradually shifting toward a modular feature-learning paradigm, which enhances the interpretability of cross-modal causal relationships and improves system iteration efficiency by decoupling complex tasks into multiple functionally independent sub-modules [34–37]. However, this approach still faces limitations: its core sub-modules often retain end-to-end learning methods, leading to the propagation and accumulation of errors across sub-modules, which ultimately compromises overall performance.

To address these challenges, we propose a new sensory-control synergetic grasping approach that can effectively transfer human grasping skills to robots, endowing robots with human-like universal grasping capability. We develop a tactile glove to capture multimodal tactile data (contact, slip and pressure) during human grasping, thereby constructing an authentic and efficient human grasping dataset. Inspired by human neurocognition on sensory information, the tactile data are fuzzily encoded into semantic grasping states (e.g. stable, slightly unstable, highly unstable) by neural-network computing, establishing a tactile-to-state mapping model. This fuzzy encoding method eliminates the uninformative dimensions of tactile signals due to deviations in grasp positions or postures across individual grabs, which greatly enhances its universality. Furthermore, we emulate a human motor strategy to build a state-to-action fuzzy controller for swiftly converting semantic grasping states to grasping actions. This human-like sensory-control synergy approach enables robots to effectively learn the experience of humans, achieving adaptive and universal grasping to diverse objects, and significantly reducing dependence on large-scale data and high-precision annotations.

## RESULTS

### Overview of human grasping skill transferring to robots

**Figure 1.**
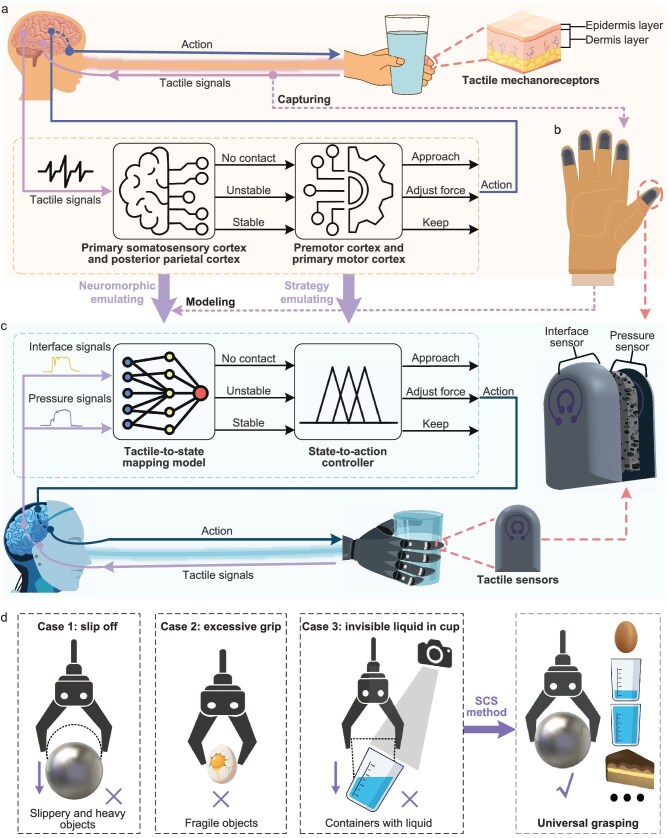
Framework of human grasping skill transfer. (a) SCS of human grasping. (b) Schematic diagram of the tactile glove equipped with tactile sensors on its fingertips. (c) SCS for robotic grasping. (d) Robotic grasping with and without SCS method in various scenarios (slippery, fragile and heavy objects).

In human grasping (Fig. 1a), tactile mechanoreceptors on fingers of the human hand perceive mechanical stimuli, such as contact, pressure and slip, and convert them into tactile signals [38]. These signals are transmitted via somatosensory afferent pathways to the central nervous system. Signals undergo initial processing and complex fusion in the primary somatosensory cortex and posterior parietal cortex [39,40]. This process abstracts low-level sensing signals into higher-level semantic grasping states, such as ‘no contact,’ ‘unstable contact’ or ‘stable contact’. Subsequently, the premotor cortex and primary motor cortex generate or refine corresponding action commands from the grasping states to modulate hand muscle activity. Specifically, if judged as a ‘no contact’ state, an approach command is issued to drive the hand toward the object; in an ‘unstable contact’ state, an increasing force command is output to enhance muscle contraction and prevent slippage; and for a ‘stable contact’ state, the current muscle force level is maintained to preserve grasp stability. This human perception-control closed loop enables dynamic adjustment of hand action, thereby achieving adaptive and universal grasping.

Inspired by the human grasping mechanism, we propose a sensory-control synergy (SCS) approach, aiming to endow robots with human-like adaptive grasping capabilities. First, we develop a multimodal tactile sensing glove (Fig. 1b), on the fingertips of which the tactile sensors are deployed to perceive interface and pressure stimuli simultaneously (details of the tactile sensor and glove are described later). The glove worn on hand synchronously captures multimodal tactile signals, including contact, slip and pressure. The pressure signals also reflect the fine-grained force modulation under human grip control. Emulating the neurocognition of the primary somatosensory cortex and posterior parietal cortex on tactile signals, the multimodal tactile signals captured by the glove are fuzzily encoded into semantic grasping states (e.g. ‘no contact’, ‘unstable contact’, ‘stable contact’) by neural-network computing (details are described later). Consequently, a tactile-to-state mapping model is built to abstract low-level tactile signals into high-level cognition of interactive states, similar to that of humans. Building on this foundation, emulating the motor cortex’s control strategy of generating adaptive action responses based on grasping states, a state-to-action fuzzy controller is further built to swiftly produce adjustments to grasping actions from these interactive states (Fig. 1c).

Finally, the developed SCS framework is deployed onto a robotic platform. The tactile sensors with the same functions as the tactile glove are deployed on the fingertips of the robotic dexterous hand. In grasping tasks, the robot continuously monitors the tactile senses and autonomously adjusts its grip actions following the human-like SCS. Experimental results demonstrate that the robotic grasping system can stably grasp diverse objects in daily life (Fig. 1d), including slippery and heavy objects (e.g. metallic ball), fragile objects (e.g. raw egg) and unstable objects (e.g. containers with liquid).

### Tactile sensing glove and human grasp sequence

**Figure 2.**
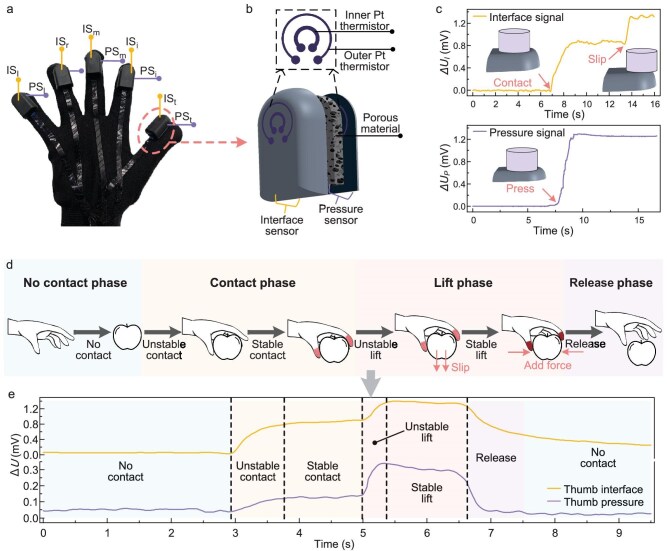
Tactile sensing and human grasp control. (a) The tactile glove equipped with tactile sensors on its fingertips. IS and PS denote interface sensor and pressure sensor, respectively; the subscripts t, i, m, r and l represent thumb, index finger, middle finger, ring finger and little finger, respectively. (b) Structure of the tactile sensor. (c) Function of the tactile sensor. (d) Sequential phases during human grasping process: no contact, contact, lift and release. (e) Thumb tactile signals captured during human grasping.

For the acquisition of tactile data in human grasping, we developed a tactile glove equipped with homemade tactile sensors on its fingertips (Fig. 2a). The tactile sensor employs a hierarchical architecture comprising (Fig. 2b): (i) a top sensing layer for interfacial sensing, and (ii) a pressure sensor composed of an intermediate porous elastomeric material layer and a bottom sensing layer [41]. Figure S1a and S1b show the fabricated prototype of the tactile sensor (fabrication is described in the Supplementary data). Each sensing layer works based on the thermo-sensation principle and monolithically integrates dual concentric Pt sensing elements on a flexible polyimide substrate, as shown in Fig. S1c. The inner Pt sensing element (ISE) on the top layer is electrically heated and generates a thermal field. It serves as a thermistor to detect interfacial states (e.g. contact or slip that induces thermal change), enabling real-time perceptions of contact and slip events. The inner Pt sensing element on the bottom layer perceives pressure stimuli by detecting the thermal conductivity changes of the intermediate porous elastomeric material layer (Fig. 2c). Under external pressure, the elastic compression of the porous material alters its effective thermal conductivity, thereby changing the heat transfer of the bottom Pt sensing element, by which the pressure is detected. The outer Pt sensing element (OSE) is unheated and functions as an ambient temperature sensor, and also eliminates the temperature interference on the inner sensing element via a differential compensation algorithm.

The sensing performances of the tactile sensor are evaluated and shown in Fig. S2. In Fig. S2a, the pressure-response curves under loading and unloading cycles (0–30 N) remain almost consistent, indicating low hysteresis of the pressure sensing (2.8%). Figure S2b highlights the sensor’s remarkable sensitivity, with a lower detection limit of <0.005 N and a fast response time of 70 ms. Figure S2c illustrates the cross-interference between the top interface sensing and the bottom pressure sensing, the results of which indicate a low cross-talk of the multimodal tactile sensor. Additionally, Fig. S2d demonstrates that the tactile sensor exhibits good stability across varying temperatures (from 30°C to 65°C). Long-term durability of the tactile sensor is evidenced by the repeatability test with 5000 loading–unloading cycles, as shown in Fig. S2e and S2f. Performance comparison with other tactile sensors is presented in Table S1. Our proposed tactile sensor exhibits multimodal perception capability (pressure, slip and temperature) with fast response, high sensitivity, low cross-talk, good temperature and long-term stability.

Armed with the tactile glove, human grasping can be recorded in real time. The human grasping process constitutes sequential multiple stages (Fig. 2d). In the no-contact phase, vision guides the hand toward the target object. Meanwhile, the finger joints preshape the grasp type based on the object feature (e.g. size and shape). Upon contact, a sudden increase in interface signal perceived by the tactile sensors triggers a dynamic adjustment in the finger joint flexion angles, enabling progressive stabilization of contact with the object. Following the stable contact, the lift phase commences. Once an object slipping is detected in the lift phase, the fingers augment the grip force to stabilize the grasping. When the object is delivered to the target location, the finger joints gradually release to place down the object.

During the human grasping process, the tactile-sensing glove worn on the human hand captures the tactile signals in real time (Fig. 2e). Based on the human neurocognition of tactile signals and motor control strategy employed in human grasping, we propose an SCS framework (details are given later). Specifically, the framework consists of a tactile-to-state mapping model that abstracts the low-level multimodal tactile data into the high-level cognition of interactive states in semantic expression (e.g. unstable, stable), and an adaptive fuzzy controller that generates the state-dependent actions (e.g. grip force adjustment) in each phase. The integrated SCS enables human robust interactive cognition and rapid adaptive grip control across diverse objects in complex scenarios.

### Bio-inspired tactile-state mapping model and fuzzy controller

Building the SCS framework of human grasping is essential and needs to be accessible and cost-efficient in practical application. Here, we propose a data-efficient learning method from human demonstrations dispensing with large-scale data and high-precision manual annotations.

**Figure 3.**
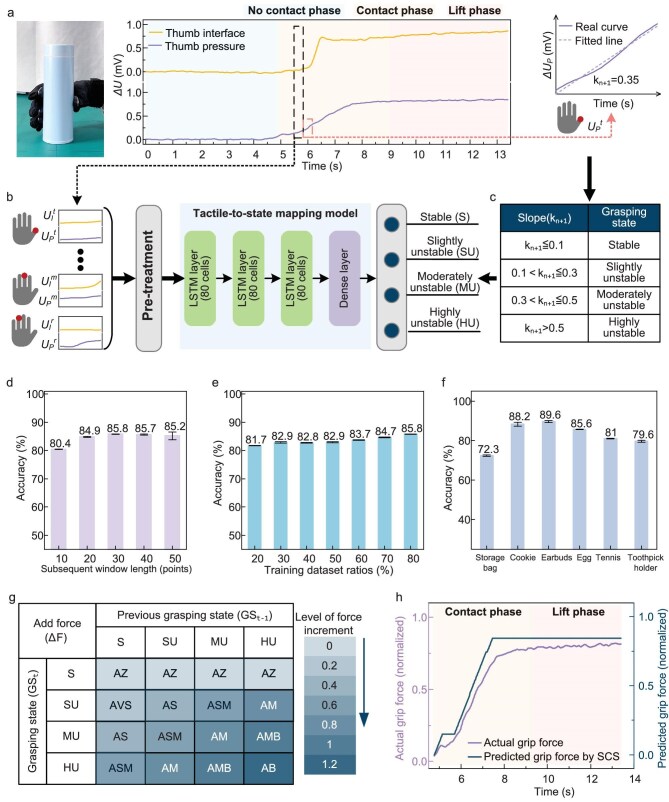
Tactile-state mapping model and fuzzy controller. (a) Thumb interface and pressure responses in human grasp of a metal cup. (b) Workflow for constructing the tactile-to-state mapping model. *U_P_*^t^ represents the thumb pressure signal, where the subscript P stands for pressure and the superscript t denotes thumb. By the same convention, the naming logic applies to other signals accordingly. (c) Criteria for partitioning states based on k_n+1_-value intervals. k_n+1_ is the slope of the fitted line for the thumb pressure response curve in the subsequent time window length. (d) State recognition accuracy with different subsequent time window lengths. (e) State recognition accuracy under different training dataset ratios. (f) State recognition accuracy for six unseen objects. The error bars are calculated from 10 grasping trials per object. (g) The strategy of state-to-action controller. AZ, AVS, AS, ASM, AM, AMB and AB represent ‘add zero force’, ‘add very small force’, ‘add small force’, ‘add small-medium force’, ‘add medium force’, ‘add medium-big force’ and ‘add big force’, respectively. (h) Normalized grip forces predicted from the SCS model and exerted by a human.

First of all, we collect the grasping dataset during human demonstrations. Wearing the tactile glove, a human grasps objects to collect the tactile datasets in the grasping processes. The datasets are utilized to build the tactile-to-state mapping model. Figure 3a presents the thumb interface and pressure signals during human grasping of a metal cup. When both interface and pressure signals generate abrupt changes with the change rates exceeding predefined threshold, the state transition from ‘no contact’ state to ‘contact’ state is triggered, thereby initiating the contact phase followed by the lift phase. Considering that the contact phase and the lift phase are kinematically delicate stages and critically dominate the grasping success, here we build the SCS framework specifically for these two phases.

Figure 3b illustrates the workflow of the tactile-to-state mapping model. Tactile signals are inherently high-dimensional time series with strong temporal dependencies. The evolution of grasp states depends not only on current sensor readings but also critically on dynamic patterns within historical signal sequences. The tactile signals first undergo preprocessing through low-pass filtering and baseline removal, followed by segmentation based on fixed time windows and normalization against signals from a standard reference object. Inspired by human neurocognition in grasping, the system fuzzily encodes multimodal tactile signals into discrete states in semantic expression, such as ‘stable’ or ‘unstable’, by neural-network computing. After comparison of data learning architectures (detailed in the Supplementary data), a long short-term memory (LSTM) network is adopted due to its demonstrated advantages in capturing long-range dependencies and modeling dynamic patterns. The details of LSTM model training are described in ‘The training details’ section of the Supplementary data and Table S2. From this, a tactile-to-state mapping model is built, which takes high-dimensional tactile signals (eight channels) as inputs and ultimately maps them into one-dimensional (1D) discrete grasping states (i.e. stable or unstable). To achieve the subsequent fine closed-loop adjustment of grip, we subdivide the ‘unstable’ state into three distinct levels: ‘slightly unstable’, ‘moderately unstable’ and ‘highly unstable’. This fuzzification processing method beneficially mitigates the interference in tactile signals during grasping, thereby significantly reducing unnecessary fluctuation in subsequent control commands and enhancing system robustness. Meanwhile, the method eliminates the need for high-precision annotation to multidimensional tactile signals, substantially lowering data-labeling costs. Furthermore, since the state-mapping model learns the associations between tactile patterns and generalized states rather than precise mappings for specific objects, it does not require retraining for new unseen objects. This significantly reduces reliance on large-scale annotated datasets and improves the model’s transferability and adaptability in complex scenarios.

As mentioned above, annotating states during grasping processes is essential for building the tactile-to-state mapping model. To reduce the burden of manual annotation, we propose an automatic labelling method. Pressure signals captured by the tactile glove record the dynamic changes of grip forces. When the current state is unstable, the brain generates a force-augmentation command to drive finger tightening, manifesting as a rising trend in subsequent pressure curves. The higher the state of instability, the greater the required grasping force increment, and the slope of the subsequent pressure signal curve increases accordingly. Based on the principle of force equilibrium, the force applied by the thumb equals the resultant force from other fingers in magnitude. Consequently, for a tactile sample within the current time window, its corresponding grasp state can be identified by the future trend of the thumb pressure curve. First, linear fitting via least squares regression is applied to the thumb pressure curve in the subsequent time window to extract the slope (k) of the fitted line (Fig. 3a). Then, partitioning states based on k_n+1_-value intervals (Fig. 3c), by which the current grasping state can be annotated from the future thumb pressure response. This annotation method enables the automatic construction of the datasets for training the tactile-to-state mapping model without any manual labeling.

Figure 3d demonstrates the average state recognition accuracies of the tactile-to-state mapping model when the thumb pressure curves with different subsequent time window lengths are used for the identification of grasping states in the grasping of 23 objects by one subject (Fig. S3a). The results indicate that the model achieves the highest state recognition accuracy when the subsequent time window length is set to 30 sampling points (at a signal sampling frequency of 50 Hz). Figure 3e illustrates the average state recognition accuracies under different training dataset ratios (defined as the percentage of training samples per object relative to its total samples) from 20% to 80%. Remarkably, with only 20% of the training data, the built model achieves an average recognition accuracy of 81.7%, significantly reducing reliance on large-scale annotated datasets (detailed in the Supplementary data). To evaluate its generalizability, the built model trained on 23 objects (Fig. S3a) is utilized to recognize the states in the grasping of six unseen objects (Fig. S3b). The results in Fig. 3f validate the strong generalizability of the model to recognize the grasping states of new unseen objects. Figure S4 shows that the model maintains high recognition accuracy, even under partial sensor failure. In cross-user evaluation, the model achieves an average recognition accuracy of 75% on the unseen user’s data, indicating its good generalizability across different users’ grasping habits (detailed experimental information is provided in the Supplementary data).

Drawing on human experience, we further design a state-to-action controller that mimics the fuzzy decision-making logic during human grasping. The controller takes 1D grasping states as inputs and outputs corresponding grip force adjustments. The control strategy is illustrated in Fig. 3g. An adaptive action occurs when humans counter instability during grasping: upon an ‘unstable’ state, the grip force is increased; if instability persists, the subsequent force increments need to exceed previous ones. This strategy enables effective suppression of instability and prevents the object from slipping or loss. Accordingly, the output of the controller depends not only on the current grasping state (GS_t_), but also on the previous grasping state (GS_t__−__1_), introducing time dependency to enhance adaptive grip capability. For instance, if the current state is ‘highly unstable’ and the previous state is ‘moderately unstable’, then the controller outputs an ‘adding medium-big force’ command. Whereas if the current state is ‘stable’, it outputs ‘maintaining current force’, i.e. an ‘adding zero force’ adjustment command. This fuzzy control with the features of state memory and graded response capabilities significantly improves the robustness and human-like nature of force adjustment in dynamic grasping environments.

The built SCS model is utilized to predict the adjustment grip force in the contact phase and lift phase of human grasping. Figure 3h exhibits that the SCS predicted grip force is almost consistent with the human exerted force during the grasping process. The result further validates the effectiveness of the SCS model for predicting human force adjustment in grasping tasks.

### Transfer human SCS to a robot

The built human SCS framework is then transferred to a robot, enabling its universal grasping. To ensure deployability on embedded systems, our framework is evaluated on a standard CPU, achieving low-latency inference suitable for real-time robotic control (detailed information is provided in the Supplementary data). It is noted that when deploying human tactile-to-state mapping model to robotic systems, considering the response latency of robots compared to humans, the system truncates the tactile signal time window. These truncated samples are extended to the model-required length via interpolation, ultimately enabling real-time grasp state judgment during robotic manipulation. The optimization of the signal window length is detailed in the Supplementary data.

**Figure 4.**
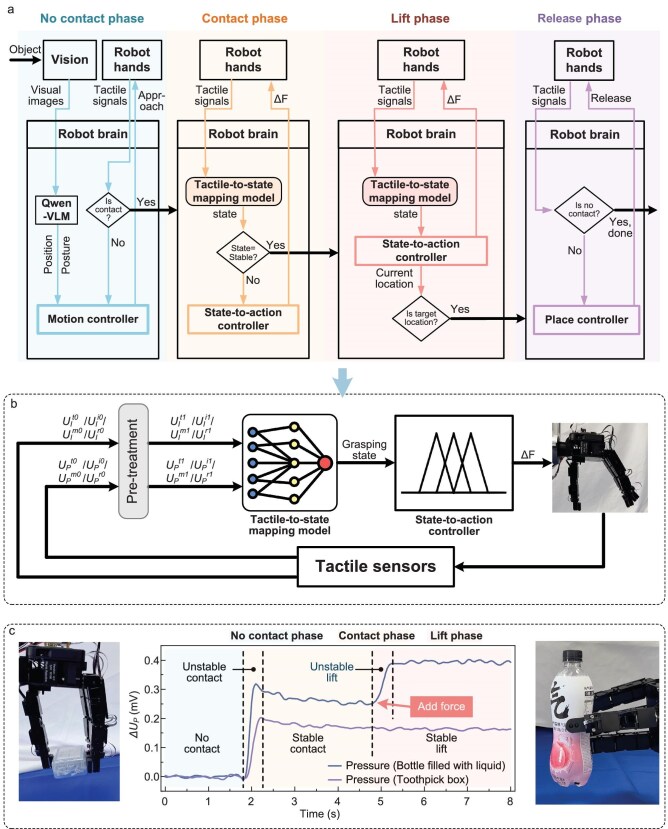
Robot with the SCS method. (a) Flowchart of robotic grasping manipulation. (b) Closed-loop workflow of SCS. *U_I_*^t0^ represents the raw interface signal of the thumb, while *U_I_*^t1^ represents the processed interface signal of the thumb, where the subscript I stands for interface sensor, the superscript t denotes thumb, the superscript 0 signifies the raw signal, and the superscript 1 denotes the processed signal. (c) Corresponding thumb pressure curves when grasping a light toothpick box and a heavy bottle filled with liquid.

Similar to the human’s grasping sequence, robotic grasping manipulation comprises four sequential phases (Fig. 4a): the no-contact phase, the contact phase, the lift phase and the place phase. Initially, in the no-contact phase, the robot captures the objects by its vision and utilizes the Qianwen visual language model (Qwen-VLM) to figure out the object’s spatial coordinates. Subsequently, the robot hand approaches and grasps the object. Upon detecting contact with the object by the tactile sensors, the robot immediately transitions to the contact phase. In this phase, the built-in tactile-to-state mapping model outputs the grasping state in real time. If the state is stable, the robot executes a lifting object action, entering the lift or delivery phase. Upon arriving at the target position, the process moves into the place phase, in which the robot gradually releases its fingers to place down the object. Figure 4b illustrates the workflow of robotic SCS in the contact and lift phase. The robotic finger-embedded tactile sensors capture the interface and pressure signals in real time. These tactile signals are fed into the built tactile-to-state mapping model, which outputs the semantic grasping states such as ‘unstable’ or ‘slightly stable’ etc. Based on this grasping state, the human-taught state-to-action controller generates adaptive force adjustment commands (ΔF). For instance, when the current grasping state is ‘slightly unstable’ and the previous grasping state is ‘moderately unstable’, the controller outputs ‘add small–medium force’ command, driving the robotic joint to further augment the grip force to enhance grasp stability.

To validate the performance of the robotic grasping, the robot with the SCS is conducted to grasp 29 objects that were previously grasped in human demonstrations (Fig. S3), with 10 grasping trials per object. The results show an average grasping success rate of 95.2% (detailed success rates per object are listed in Table S3), indicating successful transfer of grasping skill from human to robot. It is worth noting that the human SCS model is data-efficiently built, with only 20% of the dataset created by one subject. The robot with this built SCS can perceive the grasping state in real time and adaptively adjust its grip force. As shown in the thumb pressure response curve (Fig. 4c), the grasping force exerted on a lightweight toothpick box (44 g) is significantly lower than that on a heavier bottle filled with liquid (285 g). During a lift phase, the robot detects the bottle slipping (indicating an unstable state) by its tactile sensors, which triggers a force increment by the fuzzy controller. The rapid increment in grip force effectively stops the slip and achieves stable grasping of the heavy bottle. To grasp the fragile soft objects (e.g. water balloons), the robot adaptively adjusts its grip force to ensure secure and damage-free manipulation (Fig. S5).

To further validate the generalizability of robotic grasping to unseen objects, the robot is conducted to grasp another 24 objects that have never been grasped before (Fig. S6); 10 grasping trials per object. These objects have different surface textures (smooth/rough), hardness/softness, materials, shapes, sizes, weights and fragility. The results yield an average success rate of 91.25% in grasping these unseen objects (detailed results are provided in Table S4), demonstrating the good universality of the robotic grasping capability with the SCS.

**Figure 5.**
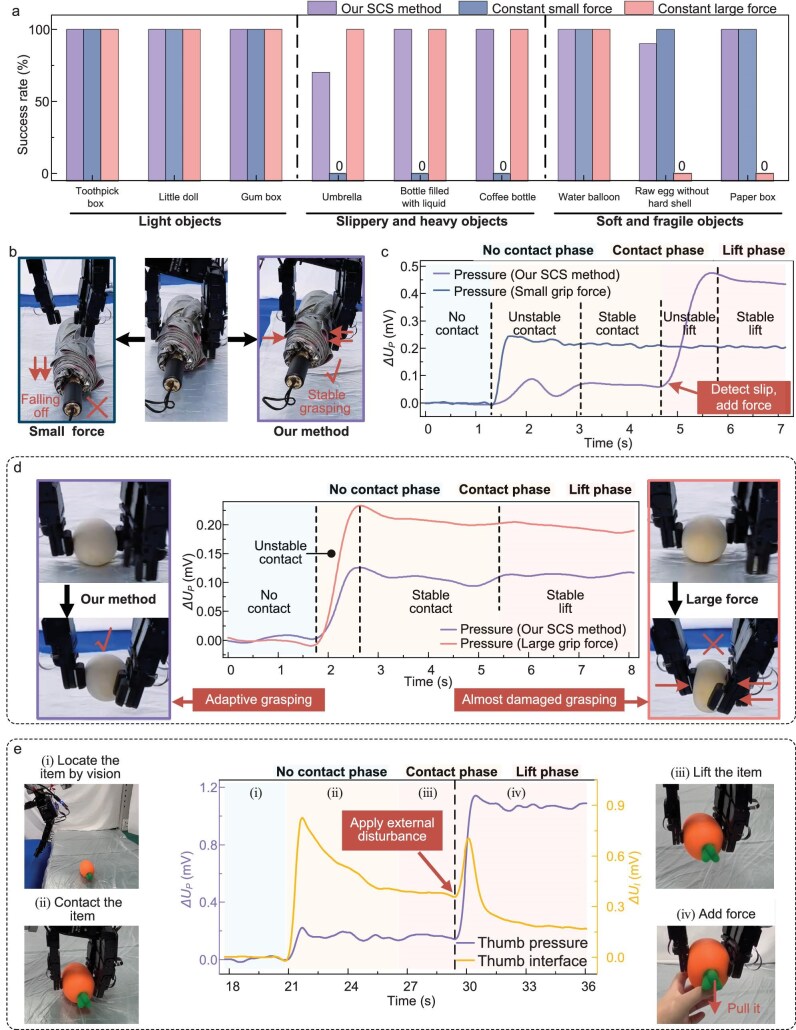
Performance of the robotic grasping. (a) Comparison of success rates between our SCS method and constant-force grasping. (b and c) Comparison between our SCS method and small grip force control when grasping a slippery and heavy umbrella. (c) Thumb pressure response signals during grasping of the umbrella. (d) Comparison with large grip force control when grasping a soft and fragile raw egg without a hard shell. Thumb pressure signals during grasping of the egg are presented. (e) Thumb interface and pressure response signals during grasping object with an external disturbance force.

To demonstrate the superiority of our robotic SCS approach over other grasping methods (e.g. constant-force control), comparative experiments were conducted to grasp slippery, fragile, soft and heavy objects. As shown in Fig. 5a, the robotic grasping with a small grip force fails to lift slippery and heavy objects, such as umbrellas (Fig. 5b and c), while a large grip force leads to damage of the soft and fragile objects. As shown in Fig. 5c, our SCS method effectively detects umbrella slipping during its lift phase and promptly increases the grasping force to ensure stable grasping. In contrast, the control method with a small grip force fails to detect the slip, resulting in falling of the object. Furthermore, as illustrated in Fig. 5d, our SCS method achieves stable and gentle grasping of a fragile raw egg (without a hard shell). Conversely, the control method with a large grip force causes severe deformation of the egg, significantly increasing damage risk. In a word, our robotic SCS method can adaptively adjust its grip force responding to different interactive states, enabling safe and successful grasping. The comparative experiments validate the human-like adaptive and generalizable grasping capabilities of our proposed SCS method.

We further validated the robotic grasping robustness in a dynamic disturbance scenario. The experiment was conducted by applying an external disturbance force in the grasping process, as illustrated in Fig. 5e. In phases (i)–(iii), the robot guides the robotic hand towards the object, and utilizes the tactile sensors on the hand to sense contact, and then lifts the object. In phase (iv), an external downward pulling force is exerted as a disturbance, and the tactile sensors detect the object slip event happening (manifested as a sudden change in the interface response curve), which triggers an adaptive force-augmentation that effectively prevents object dislodgement (Video S1).

We also evaluated the system’s performance under the conditions of sensor degradation or partial failure. Detailed experiments are described in the Supplementary data. The results (Table S5) demonstrate that the performance degradation or partial failure of the sensors has a small impact on the grasping of small-mass objects but significantly reduces the success rate in grasping large-mass objects.

### Robot accomplishes a complex task in real-world scenario

**Figure 6.**
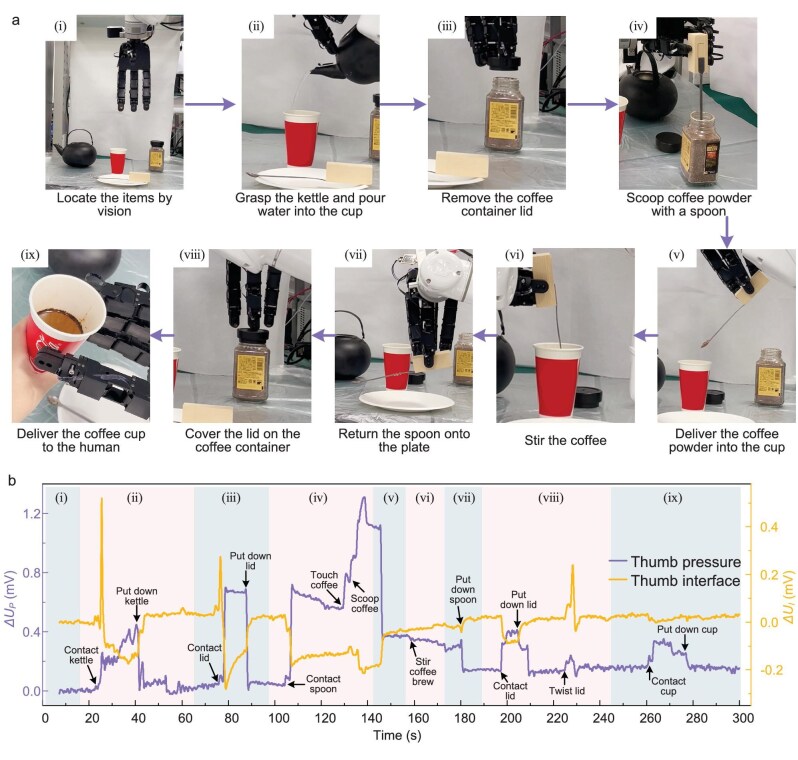
Robot coffee-brewing task. (a) The workflow of a hand-brewing coffee task. (b) Thumb interface and pressure response signals throughout the coffee-brewing process.

To show the application potential of the robotic SCS method, we applied the robot in a real-life scenario, where the robot autonomously accomplishes a task of hand-brewing coffee for a human (Video S2).The workflow encompasses nine subtasks (Fig. 6a): (i) the robot locates the items on the table; (ii)–(ix) the robot sequentially executes subtasks including grasping the kettle to pour water, opening the lid, scooping coffee powder, pouring the powder and stirring, returning the spoon, closing the lid, and delivering the coffee to the human. Notably, during coffee powder scooping, the robot utilizes the tactile sensors on the hand incorporated into the tactile-to-state mapping model to determine spoon-powder contact states, exhibiting the merit of tactile-sense in opaque container scenarios. The thumb interface and pressure signals captured in the grasping process (Fig. 6b) confirm adaptive grasping force adjustments to deal with various uncertainties in the task sequences. Table S6 shows the cumulative success rate at each step of the coffee brewing task. Experimental results evidence that the robotic system possesses human-like task generalization and environmental adaptation in complex real-world scenarios.

## DISCUSSION

In this paper, we propose a human-taught SCS approach, endowing robots with human-like adaptive and universal grasping capabilities. To address the inefficiency and complexity of data collection and decoding in dexterous manipulations, we developed a tactile glove to capture multimodal tactile signals (contact, slip and pressure) in human demonstrations, constructing authentic and efficient human grasping datasets. Inspired by human neurocognition on sensory information, the system encodes multimodal tactile data into high-level semantic grasping states by neural-network computing, establishing a tactile-to-state mapping model. This semantically encoded method eliminates the fluctuations in tactile signals due to deviations in grasp positions or postures across individual grabs, and thus enhances the validity and transferability of interactive data. Furthermore, we emulate human grasping action experience to build a state-to-action fuzzy controller for swiftly converting semantic grasping states to grasping actions.

This human-like SCS framework enables robots to achieve universal grasping across diverse objects of daily life. Experimental validation demonstrates that the robot achieves an average grasping success rate of 95.2% in grasping slippery, fragile, soft and heavy objects. The SCS approach can be efficiently built (with only 20% of the dataset created by one subject) and is highly generalizable to grasp unseen objects by emulating human neurocognition and adaptive interaction. This approach empowers robots with generalized adaptive grasping capabilities through human experience transfer, providing a promising avenue for an intelligent robot accomplishing complex tasks in real-world scenarios.

## Supplementary Material

nwaf583_Supplemental_Files

## Data Availability

The data that support the findings of this study are available from the corresponding author upon reasonable request.
