# Supplementary material for: Human-taught sensory-control synergy for universal robotic grasping
Source: Natl Sci Rev. 2025 Dec 22;13(3):nwaf583. doi: 10.1093/nsr/nwaf583 (PMC12875117; doi:10.1093/nsr/nwaf583)
Supplement: nwaf583_Supplemental_Files [file nwaf583_supplemental_files.zip › Revised Supplementary Information NSR.pdf]

Supporting Information for

Human-Taught Sensory-Control Synergy for Universal Robotic Grasping

The PDF file includes:

Materials and Methods

1) Fabrication of the sensing element
2) Fabrication of the porous material
3) Assembly of the multimodal tactile sensor
4) Preparation of the tactile gloves
5) The training details
6) Model performance metrics

Supplementary Text

1) Comparison of data learning models
2) Evaluation of model data efficiency
3) Cross-user model generalization evaluation
4) Temporal window optimization for human-robot speed discrepancy
5) System performance evaluation under sensor impairment

Supplementary Figures

Figure S1. The multimodal tactile sensor
Figure S2. Performances of the multimodal tactile sensor
Figure S3. Objects grasped by the glove
Figure S4. Performance evaluation of the model under partial sensor failure
Figure S5. Grasping a water balloon
Figure S6. 24 unseen objects
Figure S7. Comparative evaluation of classification accuracy among LSTM, CNN, and SVM models
Figure S8. 5-fold cross-validation recognition accuracy for the model

Supplementary Tables

Table S1. Comprehensive performance comparison of tactile sensors
Table S2. The detailed network structure parameters
Table S3. Grasping success rates for 29 objects
Table S4. Grasping success rates for 24 new objects unseen for the glove
Table S5. Grasping success rates under different sensor conditions
Table S6. Cumulative success rate for each step of the coffee brewing task
Table S7. The impact of signal window length on grasping success rate

Other Supplementary Materials for this manuscript include the following:

Video S1. Rapid force adjustment for stable grasping in dynamic disturbance.
Video S2. The robot autonomously accomplishes a hand-brewing coffee task.

Materials and Methods

1) Fabrication of the sensing element

The sensing element fabrication process consists of the following steps: 1) A 30 μm photoresist (KXN5735-LO, Rdmicro Co., Ltd.) is spin-coated on a polyimide substrate (AP8525R, DuPont Co., Ltd.). 2) The sensing element pattern is obtained by photolithography. 3) 30 nm thick chromium is sputtered as an adhesion layer and then 120 nm thick platinum is sputtered as the thermosensitive layer. 4) The patterned wafer is immersed in acetone for 2 h to dissolve the photoresist and then washes with absolute ethanol and deionized water. 5) The sensor is annealed at 200°C for 2 h in a vacuum oven for heat aging treatment. 6) 4 μm thick parylene film is deposited on the sensing element as a protective layer.

2) Fabrication of the porous material

The porous material fabrication process consists of the following steps: 1) Mixing the PDMS (Sylgard 184, Dow Corning Company) base agents and curing agents with a mass ratio of 10:1 to prepare the PDMS solution. 2) Silver nanoparticles (diameter < 100 nm, S110970, Aladdin Co. Ltd.), PDMS solution, and citric acid monohydrate particles (CAM) are mixed at a mass ratio of 1:5:11. The mixture is fully stirred for 10 min, then cured at 75°C for 3.5 h in a mold. 3) The cured mixture is immersed in ethanol for 24 h to dissolve the CAM to form the porous material. 4) The porous material is washed with deionized water and dried at 75°C for 1 h.

3) Assembly of the multimodal tactile sensor

The assembly of the multimodal tactile sensor includes the following steps: 1) A sensing element is adhered to a miniature printed-circuit board (PCB) substrate. 2) A porous material is adhered to the exposed surface of the sensing element, yielding a pressure sensing unit. 3) Another sensing element was positioned at the bottom of a mold. Polyurethane (PU) solution is then injected into the mold and cured at 100°C for 1 h, producing a flexible interface sensing unit. 4)The interface sensing unit is bonded onto the pressure sensing unit to form a fully integrated multimodal tactile sensor.

4) Preparation of the tactile gloves

The preparation of the tactile glove starts with a low-cost, elastic nylon glove that can be bought from various physical or online stores. The tactile sensor is then securely fixed to the exterior of the glove's fingertip, with its sensing face oriented outward for sensing.

5) The training details

The detailed network structure parameters are listed in Table S2. The model employs categorical cross-entropy as the loss function and utilizes the Adam optimizer for training.

6) Model performance metrics

The model contains a total of 132,804 parameters and requires approximately 6.57 MFLOPs of theoretical computational complexity. It is exported in ONNX format and deployed for inference using ONNX Runtime in a CPU environment. For real-world robotic control, our computing platform utilizes an Intel Core i9-11900K CPU, with ONNX Runtime optimized via the CPUExecutionProvider. In this configuration, the average inference latency is about 1 ms, while the peak CPU memory usage during inference remains around 0.34 MiB.

Supplementary Text

1) Comparison of data learning models

We conduct comparative experiments for LSTM, CNN and SVM models respectively using the same 20% training dataset. The results (Figure S7) show that the LSTM model adopted in this study achieves the best performance, with an average recognition accuracy of 82.1%, surpassing the CNN model (81.2%) and the SVM model (78.7%). In terms of the underlying mechanisms, the CNN excels at extracting local spatial features through convolutional operations, while the SVM, grounded in statistical learning theory, constructs an optimal hyperplane for classification. In contrast, LSTM is better at capturing dynamic patterns and long-term dependencies for temporal sequences. For the time-series-related task addressed in this study, the LSTM model shows advantages even under the constraint of using only 20% of the data for training, making it the superior choice for the current task.

2) Evaluation of model data efficiency

We employ a 5-fold cross-validation strategy to evaluate the data efficiency of the model, in which the grasping data for each object is evenly divided into 5 parts. In each fold, one part (corresponding to 20% of the total data) is used for training, while the remaining 80% serves as the test set. After five iterations, the model consistently achieves an average recognition accuracy above 81.7% (Figure S8), demonstrating robust performance even with limited annotated data.

3) Cross-user model generalization evaluation

We conduct a cross-user generalization experiment. A new user (User B) wears the same tactile glove and grasps 5 objects (5 trials for each): Pen holder, Chewing gum container, Storage bag, Apple, and Toothpick holder. The User B adopts different grasping postures in each grasping trial, thereby constructing a test dataset across grasping gestures. In the testing phase, the model pre-trained by using the data of a previous user (User A) is directly applied to recognize the grasping states of User B without retraining. The results demonstrate that the pre-trained model achieves an average recognition accuracy of 75% on the unseen user's data, indicating its good adaptability and generalizability to different users' grasping habits.

4) Temporal window optimization for human-robot speed discrepancy

We conduct an experiment where the robot grasps an object (a pen holder) using the SCS method under different tactile signal window lengths (5, 15, 25, 35, and 45 data points) and test the corresponding grasping success rate. The robot performs 10 grasping trials for each condition. For each window length, the tactile signals are standardized to a uniform input size via interpolation. The results (Table S7) indicate that excessively short window lengths fail to provide sufficient dynamic information from the signal sequence, leading to the state misclassification and the low success rate. With an appropriate length window, the model obtains the sufficient information to accurately recognize the grasp states,

significantly improving grasping success.

5) System performance evaluation under sensor impairment

Experiments are performed using the middle finger sensor as an example. The sensor degradation is conducted by reducing the sensor signal gain to half of its original value. The sensor failure is considered by omitting the signal (set to zero) of the middle finger sensor during grasping. For each scenario, 10 grasping trials are carried out for each of 5 objects, and the grasping success rate is used as the evaluation metric. The results are shown in Table S5.

Participants

Experiments performed in studies involving human subjects were approved by the Institution Review Board of Tsinghua University (No. 20180009). And informed consent was obtained from the human subjects to use the image and conduct the experiments described in this paper.

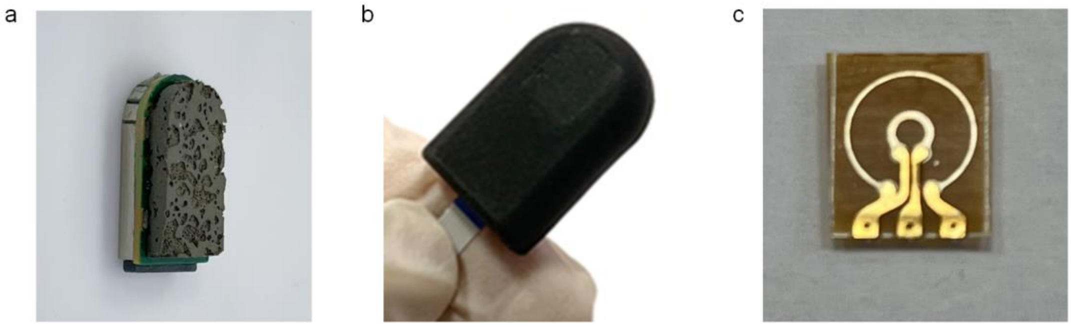

Figure S1. The multimodal tactile sensor. (a) Prototype of the bottom pressure sensor. (b) Prototype of the tactile sensor integrating the top interfacial sensing layer and the bottom pressure sensing layer (multimodal tactile sensor). (c) Dual concentric Pt sensing elements.

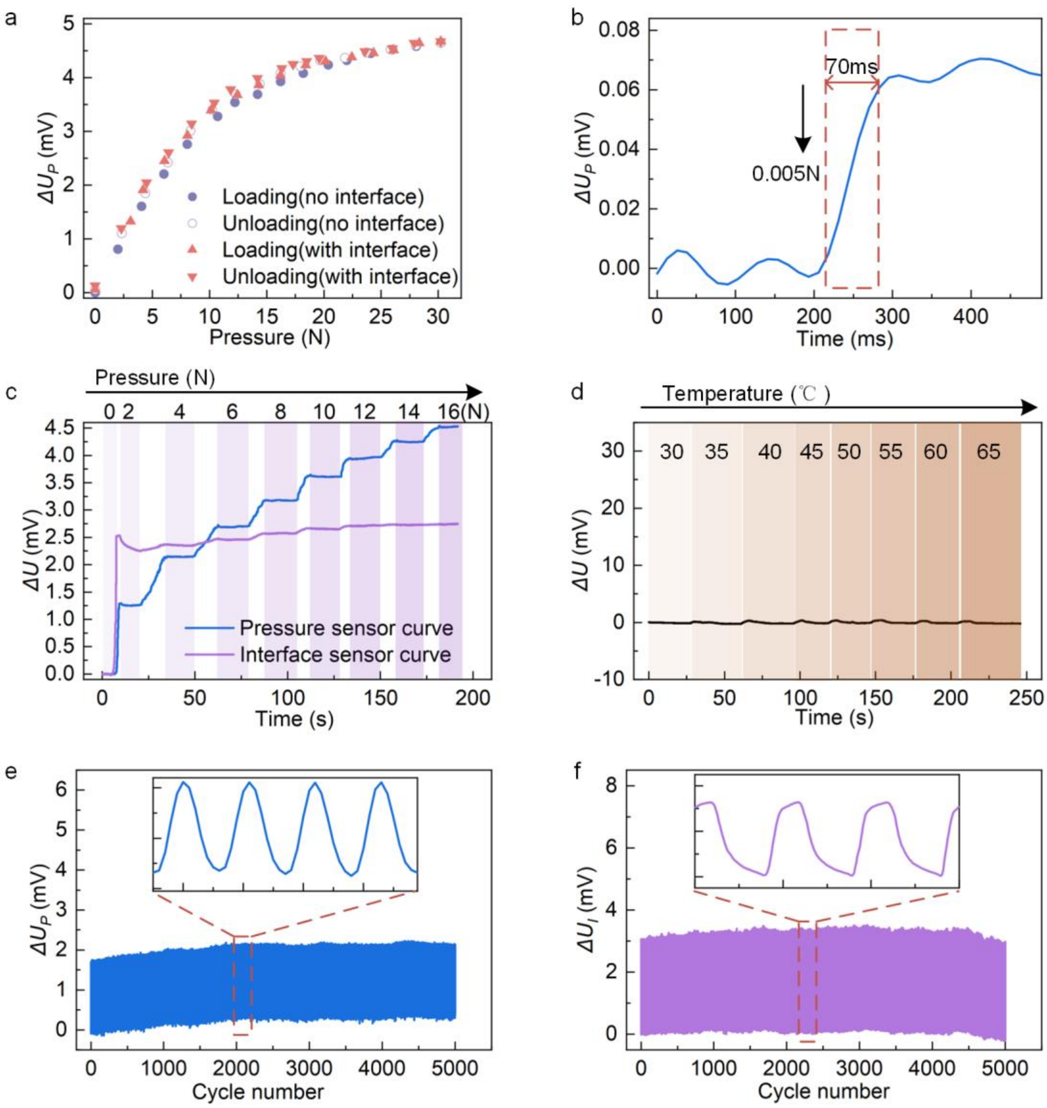

Figure S2. Performances of the multimodal tactile sensor. (a) Pressure response curves of the sensor before and after integration of the interface sensing layer. (b) Lower detection limit and response time of the sensor. (c) Interface response curve and pressure response curve under continuously applied normal force on the multimodal tactile sensor. (d) The tactile sensor exhibits good stability across varying temperatures. (e) Long-term durability of the tactile sensor, Pressure sensing. (f) Long-term durability of the tactile sensor, Interface sensing.

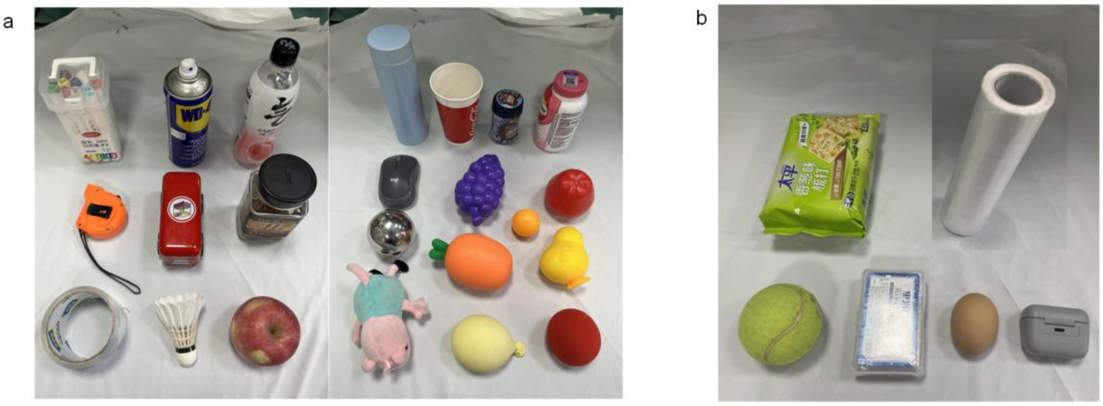

Figure S3. Objects grasped by the glove. (a) 23 objects. (b) 6 new unseen objects for the model.

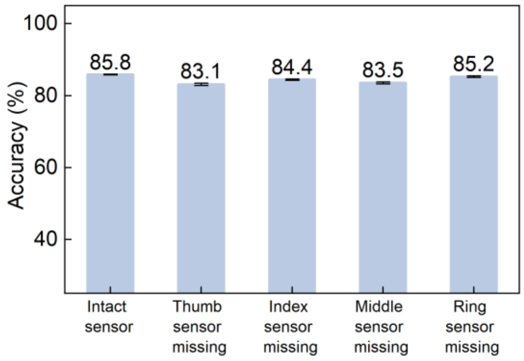

Figure S4. Performance evaluation of the model under partial sensor failure. "Intact sensors" indicates that all sensors are functioning normally. "Thumb sensor missing" indicates that signals from the thumb finger are omitted (set to zero) to construct an incomplete-input test set, and so on for the other cases.

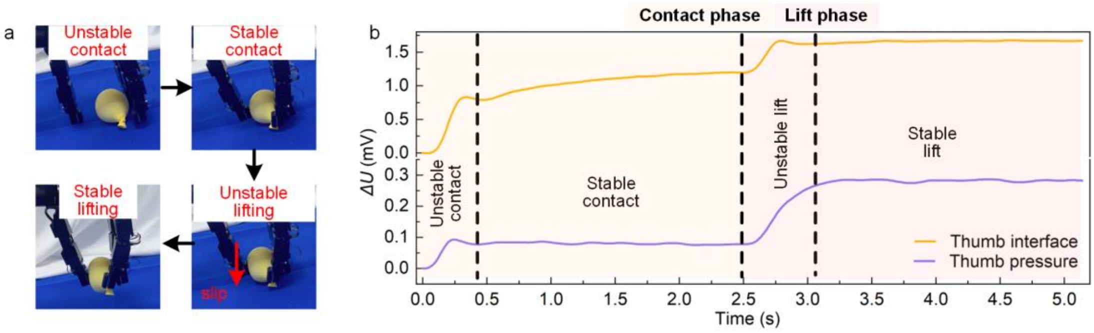

Figure S5. Grasping a water balloon. (a) Schematic of water balloon grasping. (b) Thumb interface and pressure response curves during grasping a water balloon.

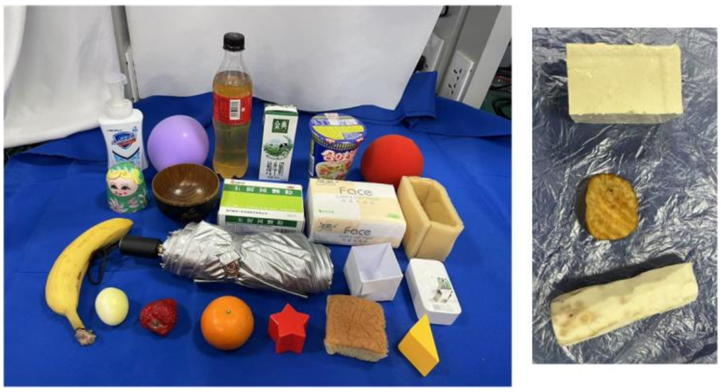

Figure S6. 24 unseen objects.

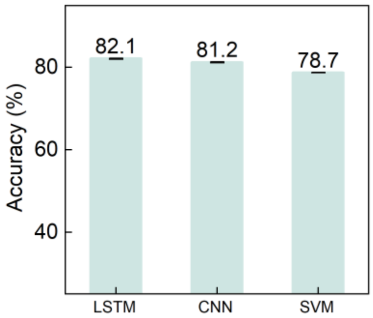

Figure S7. Comparative evaluation of classification accuracy among LSTM, CNN, and SVM models. All models are trained using the same training dataset (20% of the total data).

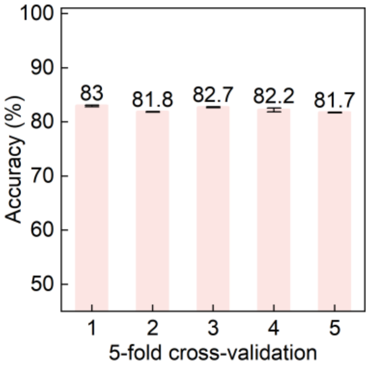

Figure S8. 5-fold cross-validation recognition accuracy for the model.

Table S1. Comprehensive performance comparison of tactile sensors

Sensor type	Force	Temp.	Slip	Full scale	Detection limit	Response (ms)	Durability (cycles)	Ref.
Piezoresistive sensor	√			0.15N	0.0054N	112	>2500	[1]
Hybrid sensor*	√	√		300kPa	–	70	>6000	[2]
Piezoresistive sensor	√			1000kPa	1.2Pa	46	2500	[3]
Capacitive sensor	√			500kPa	8Pa	40	>6000	[4]
Piezoresistive sensor	√			250kPa	2Pa	18	>20000	[5]
HEX-E QC	√			200N	0.8N	–	–	[6]
GelSight Mini	√			–	–	–	1000	[7]
XELA	√			14.7N	0.001N	–	–	[8]
**This work**	√	√	√	**30N**	**0.005N**	**70**	**>5000**	–

* Hybrid sensor (Ref. [2]) refers to a sensor that integrates multiple distinct sensing mechanisms.

Table S2. The detailed network structure parameters

No.	Layer(type)	Output Shape
1	sequenceInput	(None, 25, 8)
2	Lstm (LSTM)	(None, 25, 80)
3	Lstm (LSTM)	(None, 25, 80)
4	Lstm (LSTM)	(None, 80)
5	Dense (Dense)	(None, 4)

Table S3. Grasping success rates for 29 objects

ID	Item	Weight (g)	Success rate (%)	ID	Item	Weight (g)	Success rate (%)
1	Pen holder	232	100	2	Tennis	54	90
3	A bottle filled with liquid	285	100	4	Toothpick holder	44	100
5	Dusting canister	177	90	6	Cookie	107	100
7	Tape measure	173	90	8	Egg	52	80
9	Storage bag	242	90	10	Earbuds	47	90
11	Thermal cup	175	90	12	Empty paper cup	6	100
13	Chewing gum container	74	100	14	Pill bottle	128	100
15	Computer mouse	72	90	16	Grape model	13	100
17	Apple model	11	100	18	Iron sphere	77	70
19	Soft radish toy	92	100	20	Plush chick figurine	64	100
21	Doll	24	100	22	Water-filled orb	130	100
23	Tiny sponge ball	2	100	24	Miniature toy vehicle	85	100
25	Coffee bottle	301	100	26	Clear adhesive tape	36	100
27	Badminton	5	100	28	Apple	177	80
29	Table tennis ball	2	100		**Average success rate**		**95.2**

Table S4. Grasping success rates for 24 new objects unseen for the glove

ID	Item	Weight (g)	Success rate (%)	ID	Item	Weight (g)	Success rate (%)
1	Banana	171	80	2	Peeled hard-boiled egg	48	100
3	Orange	97	80	4	Balloon	2	100
5	Origami box	5	100	6	Strawberry	39	100
7	Cup noodles	100	100	8	Facial tissue	109	100
9	Medicine box	121	100	10	Cake	47	100
11	Triangular toy block	8	100	12	Star-shaped toy block	10	90
13	Nesting doll	39	70	14	Charger plug	94	90
15	Milk box	269	80	16	Big Sponge ball	13	100
17	Wooden bowl	55	100	18	Hand sanitizer	202	100
19	Coca-Cola beverage bottle	486	70	20	Umbrella	397	70
21	Empty foam container	7	100	22	Peeled Chinese yam	50	90
23	Potato chips	2	90	24	tofu	65	80
	**Average success rate**		**91.25**				

Table S5. Grasping success rates under different sensor conditions

ID	Item	Weight (g)	Success rate in normal condition (%)	Success rate under sensor degradation (%)	Success rate under sensor partial failure (%)
1	Pen holder	232	100	90	70
2	Chewing gum container	74	100	100	100
3	Storage bag	242	90	80	70
4	Apple	288	80	70	50
5	Toothpick holder	44	100	100	100

Note: **Normal condition** refers to the condition where tactile sensors are functioning normally. **Sensor degradation** refers to reducing the signal gain of the sensor to half of its original value. **Sensor partial failure** refers to losing the middle finger sensor signal during grasping. We conduct 10 grasping trials for each object.

Table S6. Cumulative success rate for each step of the coffee brewing task

Step No.	Operation description	Cumulative success rate (%)
1	Locate the items by vision	100
2	Grasp kettle and pour water into cup	90
3	Remove the coffee container lid	90
4	Scoop coffee powder with a spoon	70
5	Deliver the coffee powder into the cup	60
6	Stir the coffee	60
7	Return the spoon onto the plate	60
8	Cover the lid on the coffee container	60
9	Deliver the coffee cup to the human	60

Note: The cumulative success rate refers to the success rate for continuously executing the task from the first step to the current step. The overall success rate for accomplishing the entire task is about 60%. In contrast, when using a constant-force control method, the system fails to determine whether the spoon contacts the coffee powder. Consequently, it often fails at the fourth step (Scoop coffee powder with a spoon) and struggles to proceed with subsequent steps.

Table S7. The impact of signal window length on grasping success rate

No.	Signal window length of robot hand (points)	Grasping success rate (%)
1	5	20
2	15	90
3	25	100
4	35	100
5	45	100

Video S1. Rapid force adjustment for stable grasping in dynamic disturbance.
Video S2. The robot autonomously accomplishes a hand-brewing coffee task.

References

1. Zhang Y, Liu Q, Ren W *et al.* Bioinspired Tactile Sensation Based on Synergistic Microcrack-Bristle Structure Design toward High Mechanical Sensitivity and Direction-Resolving Capability. *Research* 2023; **6**: 0172.
2. Cao VA, Phan VQ, Nguyen NK *et al.* Multifunctional tactile sensor with multimodal capabilities for pressure, temperature, and surface recognition. *Nano Energy* 2025; **136**: 110706.
3. Wang S, Fan X, Zhang Z *et al.* A Skin-Inspired High-Performance Tactile Sensor for Accurate Recognition of Object Softness. *ACS Nano* 2024; **18**(26): 17175-84.
4. Guo X, Hong W, Liu L *et al.* Highly Sensitive and Wide-Range Flexible Bionic Tactile Sensors Inspired by the Octopus Sucker Structure. *ACS Appl Nano Mater* 2022; **5**(8): 11028-36.
5. Zhao X-H, Lai Q-T, Guo W-T *et al.* Skin-Inspired Highly Sensitive Tactile Sensors with Ultrahigh Resolution over a Broad Sensing Range. *ACS Appl Mater Interfaces* 2023; **15**(25): 30486-94.
6. OnRobot. HEX-E/H QC Datasheet. HEX-E/H QC (10 November 2025, date last accessed)
7. GelSight. GelSight Mini Product Sheet. GS_Mini_Product_Sheet_10.07.24.pdf (10 November 2025, date last accessed)
8. XELA Robotics. XELA Robotics Product Catalog for 2025. XELA Robotics - Catalog 2025.pdf (10 November 2025, date last accessed)
